# On the carrying capacity of the bone marrow survival niche in mice

**DOI:** 10.3389/fimmu.2025.1706810

**Published:** 2025-11-20

**Authors:** Keisuke Tonouchi, Chen-Hao Yeh, Derek W. Cain, E. Ashley Moseman, Barton F. Haynes, Kshitij Wagh, Kevin Wiehe, Tomohiro Kurosaki, Garnett Kelsoe

**Affiliations:** 1 Department of Integrative Immunobiology, Duke University, Durham, NC, United States; 2 Department of Medicine, Duke University, Durham, NC, United States; 3 Duke Human Vaccine Institute, Duke University, Durham, NC, United States; 4 Laboratory of Lymphocyte Differentiation, WPI Immunology Frontier Research Center, Osaka, Japan; 5 Center for Infectious Diseases Education and Research, Osaka University, Osaka, Japan; 6 Laboratory for Lymphocyte Differentiation, RIKEN Center for Integrative Medical Sciences, Yokohama, Japan; 7 Department of Surgery, Duke University, Durham, NC, United States

**Keywords:** bone marrow, plasmacyte, germinal center (GC) B cell, survival niche, durable antibody

## Abstract

Plasmacytes, the effector arm of humoral immunity, produce sufficient amounts of specific antibodies to provide protection against infection or disease. The durability of this humoral protection depends on the generation of long-lived plasmacytes (LLPC), a specialized population that is capable of secreting antibody over long periods of times - years to decades. Here we investigate the role of constitutively active germinal centers (GCs) in generating the plasmacytes resident in bone marrow, a site critical for vaccine-induced LLPC to provide meaningful protection to infection and resistance to morbidity. In unimmunized B6.S1pr2-Cre mice, we show that a short period of conditional labeling marks 85% of gut-associated GC B cells and their progeny. Frequencies of labeled GC B cells fall over time, but frequencies of labeled bone marrow PC increase to approximately one-third of all bone marrow PC by 70-80 days after pulse labeling. Labeled, GC derived bone marrow PC express the identical isotypic distribution of the unlabeled PC in bone marrow. We conclude that the progeny of gut-associated GC B cells are responsible for most, and perhaps all, bone marrow PC and that under homeostatic conditions, serum antibody reflects exposure to gut antigens. Bone marrow occupancy by these gut-derived PC raises the possibility of competition with more transient, vaccine-induced humoral responses.

## Introduction

Plasmacytes (PCs), the effector arm of humoral immunity, produce quantities of antigen-specific antibody (Ab) that protect against infection and disease; Ab persists only as long as its producing PCs survive. Most newly generated PCs are short-lived (SLPCs), but a subset of SLPCs matures further into long-lived PCs (LLPCs). LLPCs are a specialized, non-proliferating population that continuously produce high-affinity Abs for years to decades, and in some cases for a lifetime, thereby ensuring durable humoral immunity and providing critical resilience and therapeutic benefit (1, 2). LLPCs commonly reside in the bone marrow (BM), distant from their site of generation (3), where specialized niches sustain their survival and continuous Ab production (4–7). Other tissues, including spleen and gut-associated lymphoid tissues (GALT) also contain survival niches, and contribute to Ab persistence (4, 8).

In contrast, SLPCs predominate in secondary lymphoid tissues or sites of inflammation, where they produce an early and (generally) lower-affinity Ab on antigen exposure or innate signaling (9, 10). SLPCs are also abundant in BM, but most undergo apoptosis within days to weeks (10–12). These evanescent SLPCs initially produce IgM and shortly thereafter, switch to IgG (13). Their early Abs are typically encoded by unmutated V(D)J gene rearrangements and are often clonally related to sister B cells that later enter GCs (14–16).

**Figure 1:**
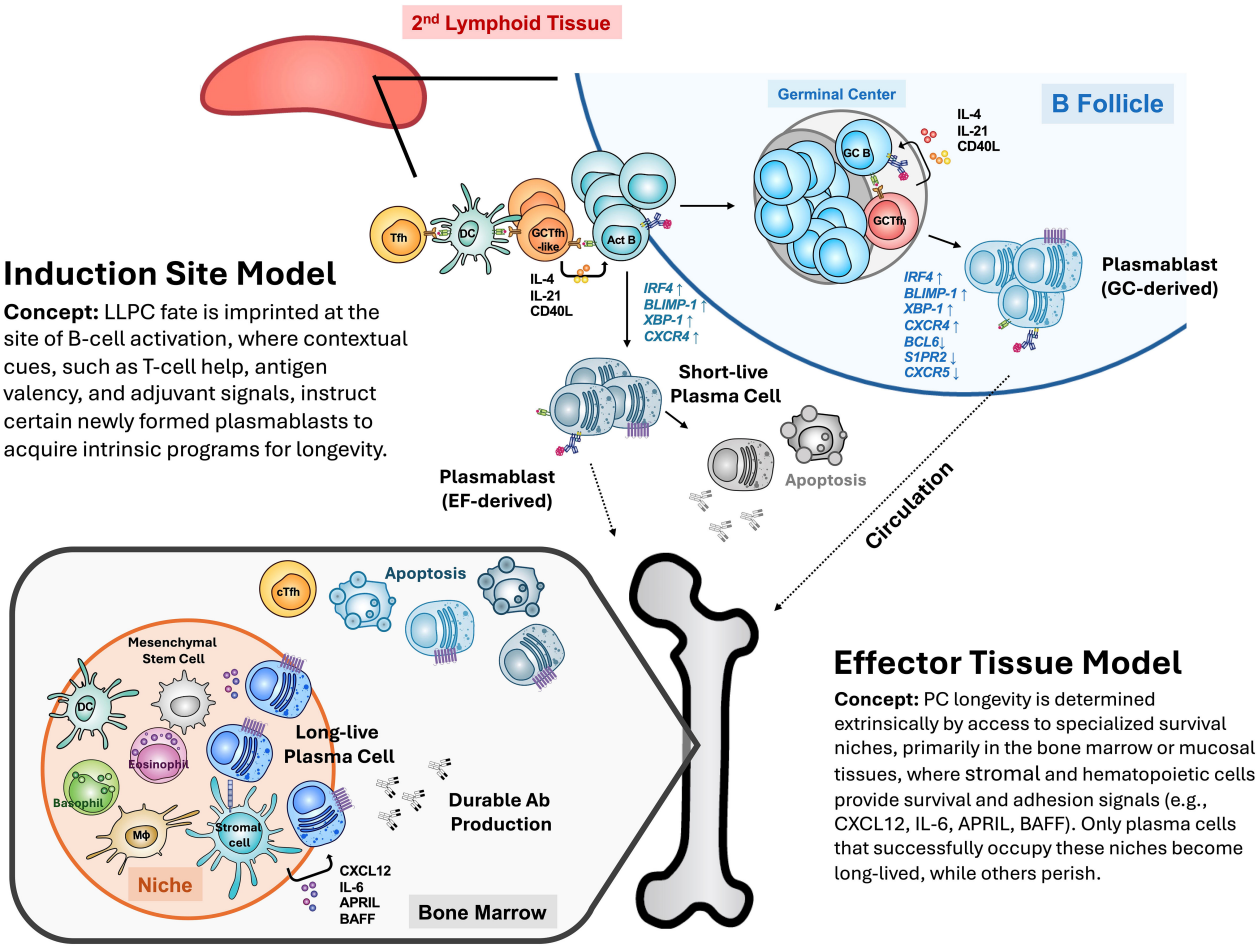
Two, non-exclusive, general models to explain the acquisition of longevity by PCs. First, the *induction site model* in which fate heterogeneity in newly generated PCs is shaped or determined by different signals or contextual cues present at the time or site of their activation (*e.g*., T-cell help, antigen-form, adjuvant properties). Alternatively, the *effector tissue model* posits that LLPC fate is determined primarily by occupancy and competition for specific survival niches established by a microenvironment of *non-lymphoid* cells within the BM, GALT or other lymphoid organs. The *induction site model* is an instructive model whereas the *effector tissue model* is a competition model.

From these observations, two, non-exclusive, general models are accepted to explain the acquisition of longevity by PCs (**Figure 1**). First, fate heterogeneity within newly generated PCs is shaped by different signals or contextual cues present at the time or site of their activation (*e.g*., T-cell help, antigen-form, adjuvant properties, *etc*.; the *induction site model*), which posits that these factors *instruct* LLPC maturation. For example, repetitive antigens elicit more durable protective humoral responses than do non-repetitive antigens (17). Alternatively, the LLPC fate is determined primarily by occupancy and competition for specific survival niches established by a microenvironment of *non-lymphoid* cells (the *effector tissue model*) within the BM or other organs (18). Indeed, the heterogeneity of niche cells was recently demonstrated (19, 20). Evidence indicates LLPCs can arise from the T cell-dependent plasmablasts (PBs) formed in germinal centers (GCs) (21, 22) and from T cell-independent humoral responses (23).

If BM SLPC and LLPCs arise most often as a result of GC responses (2, 7, 24) these PC should exhibit the spectrum of V(D)J mutations typical of GC B cells recovered from GALT (25–28). Some fraction of GC B cells, often those with higher BCR affinities, exit the GC with a commitment for PC differentiation (21, 29–31). To leave the GC, these cells down-regulate S1pr2 and CXCR5 and increase CXCR4 expression to facilitate homing to and retention in BM (32, 33). This differentiation is governed by a complex transcriptional program (34) with the transcription factor IRF4 required to initiate PC differentiation, and IRF4 expression level controlled reciprocally by reductions in BCL-6 (35–37). Subsequently, BLIMP-1 acts to repress genes essential for B-cell identity and enforce terminal PC fate (38, 39). To accommodate the intense protein-folding burden imposed by robust antibody secretion, PCs rely on the transcription factor XBP-1 to mitigate ER stress (40, 41).

Thus, the *induction site model* must accommodate multiple routes to LLPC maturation, whereas the *effector tissue model* can parsimoniously explain LLPC generation from both T-dependent and T-independent responses (6).

Despite much effort, however, the precise identity of the cells and microenvironment comprising the LLPC survival niches remains elusive and the critical interactions between SLPC and niche cells/factors that drive LLPC maturation are unknown (42, 43). Moreover, the pathway by which SLPC mature into LLPC is not understood fully, and no strategy has yet succeeded in selectively eliciting LLPC to disparate antigens. A better understanding of these biological processes will offer fundamental insight into the nature of B-cell differentiation and, perhaps, improve vaccine efficacy by increasing the durability of humoral protection by systemic Ab.

In this article, we will first examine the evidence for specialized BM niches, then consider models of BM PC turnover, discuss systemic Ab homeostasis and quantitative requirements, and finally evaluate the contribution of constitutive gut GCs.

### LLPC survival niches in BM

The importance of niche cells for maintaining serum Ab durability has been supported by studies using tumor necrosis factor (TNF) treatment and various infection models. TNF can disrupt the BM niche and, in a PC-extrinsic manner, mobilize pre-existing LLPCs from the BM, thereby diminishing pre-established serological memory (44, 45). Similarly, infections with measles virus (46, 47) or malaria (48, 49) led to reduction in pre-existing antibodies, presumably by disrupting niche cells and/or their interactions with LLPCs (46–48). From the viewpoint of the niche model, the most fundamental question remains: how is LLPC specification developed and maintained through interactions with niche cells?

In regard to physical interaction between PCs and niche cells, a recent study showed that polyclonal LLPCs, timestamped 60 days before, were less motile than total BM PCs, 80% of which are derived from SLPCs (50), consistent with observations on LLPCs specific for the nitrophenylacetyl hapten, NP (4). Because of higher expression of CXCR4 in BM LLPCs than SLPCs (50), Fooksman and his colleagues propose that the CXCL12-CXCR4 axis regulates LLPC immobilization and retention, thereby controlling LLPC specification (7). As products of GC reactions and somatic evolution, LLPCs continuously secrete high-affinity, class-switched Abs, providing a stable foundation for durable immunity (21, 51, 52). Unlike SLPCs, which display phenotypic markers associated with recent differentiation and proliferation, along with high metabolic activity, the remarkable longevity of LLPCs is not thought to be established in a cell-intrinsic manner but rather dependent on sustained survival cues from the BM microenvironment (1, 53, 54).

Candidates for this specialized niche include stromal cells, mesenchymal stem cells, eosinophils, megakaryocytes, basophils, macrophages/monocytes, and dendritic cells (55–61). These accessory cells supply a core set of chemokines (CXCL12), survival factors—IL-6, APRIL, and BAFF—and present the adhesion ligands VLA-4 and LFA-1 to anchor LLPCs within the niche. Stromal and mesenchymal cells provide CXCL12 to guide homing (32, 62, 63). Beyond its established role as a chemoattractant for LLPCs, the chemokine CXCL12 orchestrates the architecture of the niche by mediating the co-localization and retention of hematopoietic cells. These hematopoietic cells function as essential accessory components, providing a suite of survival factors including IL-6, APRIL, and BAFF (56, 61, 63–66). The latter two TNF family ligands engage their cognate pro-survival receptors, BCMA and TACI, on PCs to deliver anti-apoptotic signals (67, 68). Notably, a recent study suggests that BCMA may be dispensable for IgG-secreting LLPC maintenance under some conditions, suggesting the existence of functional differences between TACI and BCMA effects (69). Indeed, the factors regulating niche occupancy may well differ by organ and PC isotype (66). While the specific role for eosinophils in regulating BM PC remains unclear (56, 70), substantial evidence suggests that PC niches contain a supportive myeloid cell network. Ly-6C^hi^ monocytes and macrophages contribute by remodeling the extracellular matrix, clearing apoptotic debris, and providing additional IL-6 (and possibly APRIL), while basophils and dendritic cells further augment the cytokine milieu (55, 58–60).

Within this environment, LLPCs down-regulate proliferative gene programs while up-regulating anti-apoptotic factors such as Mcl-1 and other Bcl-2 family members (71). The short half-life of Mcl-1 necessitates continuous synthesis, presumably in response to constant APRIL/BAFF survival cues. To meet the demands of continuous Ab secretion, LLPCs adopt specialized metabolic strategies, including a reliance on autophagy and a high mitochondrial respiratory capacity (72). Moreover, to ensure immune homeostasis, the system employs negative feedback loops. LLPCs express the inhibitory receptor FcγRIIB, which can trigger apoptosis when cross-linked by high concentrations of antibody-antigen complexes, thereby allowing serum antibody levels to regulate the size of the PC pool (73). Considering that IgM, IgA, and IgE PC both express membrane-bound and secrete soluble Ig (74, 75), the crosslinking of FcγRIIB by membrane IgM/IgA/IgE antigen complexes might induce apoptosis in an antigen-specific manner.

Integrin expression is central to both the retention and survival of LLPCs. VLA-4 and LFA-1 bind stromal VCAM-1 and ICAM-1, respectively, mediating niche adhesion and initiating “outside-in” signaling through FAK, PI3K–Akt, and MAPK pathways that enhance resistance to apoptosis (55, 57, 65, 76, 77). In parallel, CXCL12-CXCR4 interactions engage G-protein-coupled receptor signaling that activates downstream effectors, including PI3K, Akt, and MAPK, contributing to LLPC positioning and survival (32). Together with leukocyte-derived APRIL and BAFF (activating NF-κB) and IL-6 (via JAK-STAT3), these integrin and chemokine pathways form a tightly regulated network that anchors LLPCs, delivers sustained anti-apoptotic cues, and fulfills the metabolic demands of lifelong antibody production (56, 57, 64, 67, 78).

One of us (T.K.) has recently observed that immobilization of LLPCs was correlated to cell quiescence (assessed by p27) and increased survival activity. Hence, we propose that increased mechanical interaction between PCs and adjacent niche cells may permit SLPCs to enter the G_0_ state in addition to receiving APRIL and BAFF survival factors, thereby acquiring the LLPC fate.

### On BM PC turnover

Radbruch and colleagues’ influential model for LLPC biology (24) proposed that newly generated SLPC eventually displace senescent LLPC from BM survival niches and thereby enter the LLPC compartment. This model is attractive, as it could explain the progressive changes in serum Ab specificity over time in response to novel antigen encounters. Assuming that all niches are equally capable of sustaining PCs and that all PCs are equally capable of accessing niches, Radbruch’s model predicts that diminished PC generation by anti-CD20 treatment should slow the rate of the decay of pre-established Ab titers. In practice, however, this outcome has generally not been observed (79–81), although DiLillo et al. showed that CD20^+^ cells were required to replenish BM PCs after their forced mobilization from the BM (80).

Taken together, these data suggest that the simple displacement model is insufficient. *A priori*, there is no firm reason to assume that all PC survival niches are equivalent or that all SLPCs share equal capacity to mature into LLPCs. Instead, variation in the intrinsic properties of PCs and in the supportive capacity of niche cells might provide a more refined explanation for the available experimental data.

In contrast, Amanna and Slifka proposed that antigen can imprint a lifespan onto PCs at their formation, meaning that their survival would in part reflect an internal clock that would be created at the production phase in the induction site (82). This *induction site model* for LLPC differentiation is consistent with empirical observations on the capacity of some, but not all, antigens to elicit durable Ab responses. More recently, they further suggested that a combination of T-cell help and epitope multivalency optimally promotes durable humoral responses largely independent of adjuvant choice, and that natural infections generally result in more sustained immunity (17).

Building on the niche model, subsequent studies have revealed that migration to and retention within the BM are regulated by more complex mechanisms than the original CXCL12-CXCR4 framework. For instance, PCs carrying a CXCR4 gain-of-function mutation were abundant in the spleen but barely detected in the BM, suggesting that only an optimal range of CXCR4 activity permits successful homing to the BM (83). Additional regulators, including S1PR1, CXCR3, and CD11b, have also been implicated in controlling PC trafficking and localization (18, 84). Retention requires not only adhesion molecules, as shown by the effect of anti-VLA4 blockade (80, 85), but also downregulation of S1PR1 and upregulation of its antagonist CD69, although the timing and regulation of these changes during PC maturation remain unclear (18).

A key unresolved issue is how structural CXCL12-abundant reticular cells are integrated with distinct survival-factor–producing cells such as eosinophils, megakaryocytes, and myeloid cells. Rather than a uniform niche equally accessible to all PCs, LLPC persistence may depend on dynamic and heterogeneous arrangements of these components (42). This framework stands in contrast to the *induction site model*, which posits that PC lifespan is largely predetermined at the time of their formation (82).

### Numbers of BM PC and systemic Ab

SLPC and LLPC in BM are responsible for the homeostatic levels of serum IgG and IgA in naïve mice and for the maintenance of protective Ab levels elicited by infection or immunization. Given that the LLPC population necessarily resides in survival niches, the occupancy, capacity and stability of the survival niche must control serum Ig concentrations. To a significant extent, serum IgG and IgA levels in mice are determined by incidental exposure to microbial antigens, especially by the postpartum development of gut flora (86, 87). Serum IgM levels are little affected by the absence of exogenous antigenic exposure (86). This “natural IgM” is the product of B1 or yolk sac-derived B-lineage cells; natural IgM is constitutively secreted and acts innately against the dissemination of infectious particles via low affinity interactions and efficient complement activation (88). Although natural IgM in serum is tightly linked to a population of BM PC that express membrane IgM, whether these innate-like B cells require survival niches is unknown/unclear (88). It is clear, however, that some fraction of IgM secreting BM PC originate from GC responses (see below).

If serum Ab concentrations at homeostasis are maintained by LLPC populations, then the numbers of PC secreting IgM, IgG, or IgA can be estimated. For example, to estimate the population size of PCs needed to support resting serum IgG levels in mice, we begin with the rate equation describing the total number of serum antibodies, $N_{A}$, which can be expressed as Equation 1:

(1) $$\frac{dN_{A}}{dt}=\;-\lambda N_{A}+rN_{P}$$

Where λ is the decay rate of antibodies, r is the rate of antibody production, and $N_{P}$ is the total number of PCs.

At steady state, $\frac{dN_{A}}{dt}=0$, and:

(2) $$N_{P}=\;\frac{\lambda N_{A}}{r}$$

The antibody decay rate λ is related to the antibody half-life, $t_{1/2}$ by:

(3) $$\lambda =\frac{\text{ln}(2)}{t_{1/2}}$$

Substituting Equation 3 into 2:

(4) $$N_{P}=\frac{\text{ln}(2)\;N_{A}}{r\;t_{1/2}}$$

The total serum lg concentrations in adult C57BL/6 mice range from 2–3 mg/ml with isotype specific serum levels of: IgM, 0.22 (± 0.09) mg/ml; IgG1, 0.28 (± 0.09) mg/ml; IgG2c, 1.22 (± 0.35) mg/ml; IgG3, 0.18 (± 0.08) mg/ml and; IgA, 0.075 (± 0.025) mg/ml (89). Combining all IgG classes, total serum IgG concentration is 1.68 mg/ml.

The total blood volume of a 25g mouse is about 1.96ml (90, 91), and the serum volume is approximately 0.98 ml. Thus, total serum IgG [(1.68 mg/ml) x (0.98 ml)] has a mass of about 1.65 mg. The average molecular mass of mature, glycosylated IgG is 155 kDA, corresponding to an estimated 3.88 x 10^15^ molecules of IgG/mg. Therefore, the estimated number of IgG molecules in the total serum of a mouse is 6.39 x 10^15^.

The half-life of mouse IgG antibodies is estimated at ~216 hours (92, 93). Using the range of secretion rates measured for a mixed population of human BM antibody-secreting cells (short-lived: CD19^+^IgD^-^CD38^hi^CD138^-^; intermediate: CD19^+^IgD^-^CD38^hi^CD138^+^; and long-lived: CD19^-^IgD^-^CD38^hi^CD138^+^) (94), we estimate the mouse BM PC secretion rate as between 1527 and 2829 IgG molecules/s.

Inputting these estimated values of IgG half-life, antibody secretion rate, and total number of IgG molecules in the serum into Equation 4, we estimate that maintaining steady-state IgG levels requires between 2.0 and 3.7 million PCs.

**Figure 2:**
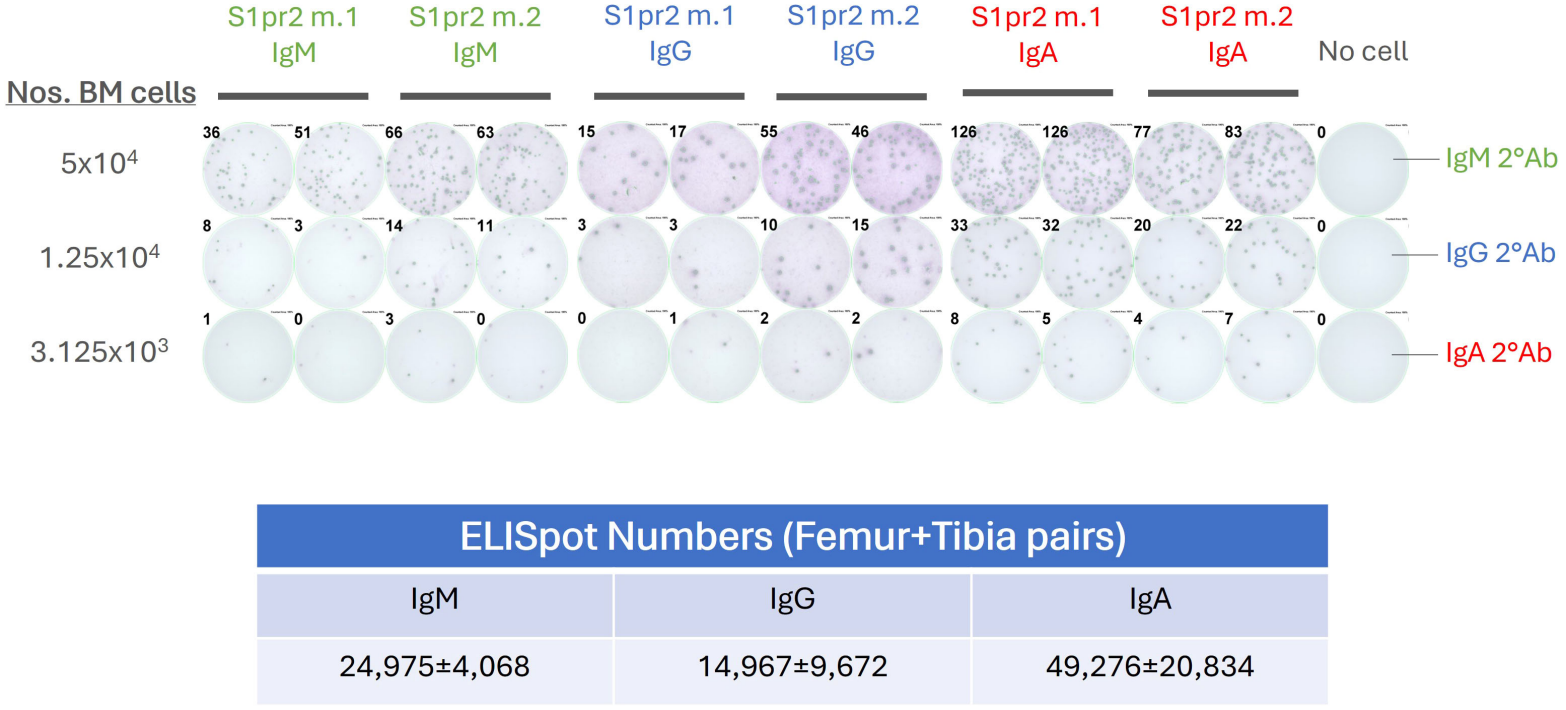
ELISpot assay on different Ig secreting cells in BM. BM cells were applied in serial, four-fold dilutions (5.0x10^4^ – 3.1x10^3^) onto the membrane of ELISpot plates and incubated for 1 hour at 37°C. Spots captured by anti-Igκ and -Igλ antibodies on membrane were visualized by AP-conjugated secondary antibodies and counted using a CTL ImmunoSpot S6 instrument. Assay was done by using two animals in duplicate manner. Raw images of individual wells are shown on top with the counts at the top left of each. Far right column shows the wells without any cells but incubated with each anti-Ig secondary antibodies. Bottom table represents the mean±SD number of each Ig secreting cells in femur+tibia pairs of BM calculated by the ELISpot counts.

Analyses of antibody secreting cells (ASC) in BM by ELISpot are consistent with these flow cytometry data. BM cells were recovered from femur and tibia pairs from unimmunized B6.S1pr2 mice (n=2) and plated in serial, four-fold dilutions (5.0x10^4^ – 3.1x10^3^) onto ELISpot membranes (96-well plate). Following 60 minutes of incubation, ASC secreting IgM, IgG, or IgA counted in a CTL ImmunoSpot S6 instrument (**Figure 2**). In this way, we observed 24,975 (± 4,068) IgM ASC, 14,967 (± 9,672) IgG ASC, and 49,276 (± 20,834) IgA ASC. As femur/tibia pairs represent about 19% of central BM volume (95), we estimate that the BM supports some 129,000 IgM ASC, 77,000 IgG ASC, and 254,000 IgA ASC. The BM of unimmunized mice contains approximately 470,000 SLPC and LLPC with 28% secreting IgM, 17% IgG, and 55%, IgA (**Figure 2**). We propose that this represents the baseline capacity of the BM to support SLPC and LLPC and defines the extent of BM survival niche in homeostasis.

If the capacity of the BM survival niche in homeostasis is approximately 500,000 SLPC and LLPC (**Figure 2**), it cannot be easily reconciled with our estimate for the numbers of IgG secreting PC required to maintain steady-state serum IgG concentrations. Even for a *back-of-envelope* calculation, the result is too far off - by a factor of ≥25 - to explain PC numbers necessary for steady-state IgG levels (79,000 vs. 2,000,000 PC). Several possible errors in these calculations and enumerations are obvious: longer IgG half-lives, higher PC secretion rates, incomplete recovery of BM PC, *etc*. Regardless of the causes of this discrepancy, the issue of niche capacity is critical to understanding what is possible for humoral immunity elicited by infection or immunization. Another, and likely, possibility is that PCs residing in survival niches outside the BM contribute substantially to serum antibody titters. Supporting this possibility are recent animal and human studies identifying LLPC populations within the gut and intestine (4, 8, 96). These results also suggest that the entire niche capacity for IgG PC is not very large, even if it encompasses two million cells. For example, consider an IgG LLPC repertoire elicited by 200 disparate and complex antigens, with each antigen bearing 10 discrete epitopes. If equally distributed, an LLPC compartment of 2,000,000 IgG ASC would be composed of 2,000 (200 x 10) LLPC epitopic groups of 1,000 cells each. This would represent a stable expression of only about 0.8μg/ml serum for IgG Ab to each epitope; BCR diversity within individual epitopic groups would further reduce concentrations of a specific circulating IgG Ab.

### Constitutive GCs and competition for BM niches

Germ-free mice display impaired development of gut-associated lymphoid tissues, characterized by fewer and smaller Peyer’s patches and mesenteric lymph nodes (MLNs) compared with conventionally raised controls. These underdeveloped lymphoid tissues also contain reduced numbers of GCs (97). Moreover, germ-free mice exhibit ~95% reductions in circulating IgG and IgA relative to controls, despite otherwise normal B cell development (86, 98). Taken together, these data imply that GC responses elicited by normal gut flora are largely responsible for homeostatic levels of serum IgG and IgA in laboratory mouse strains. Consequently, the BM survival niches necessary for LLPC persistence are occupied by the progeny of GC B cells arising in mucosal lymphoid tissues.

**Figure 3:**
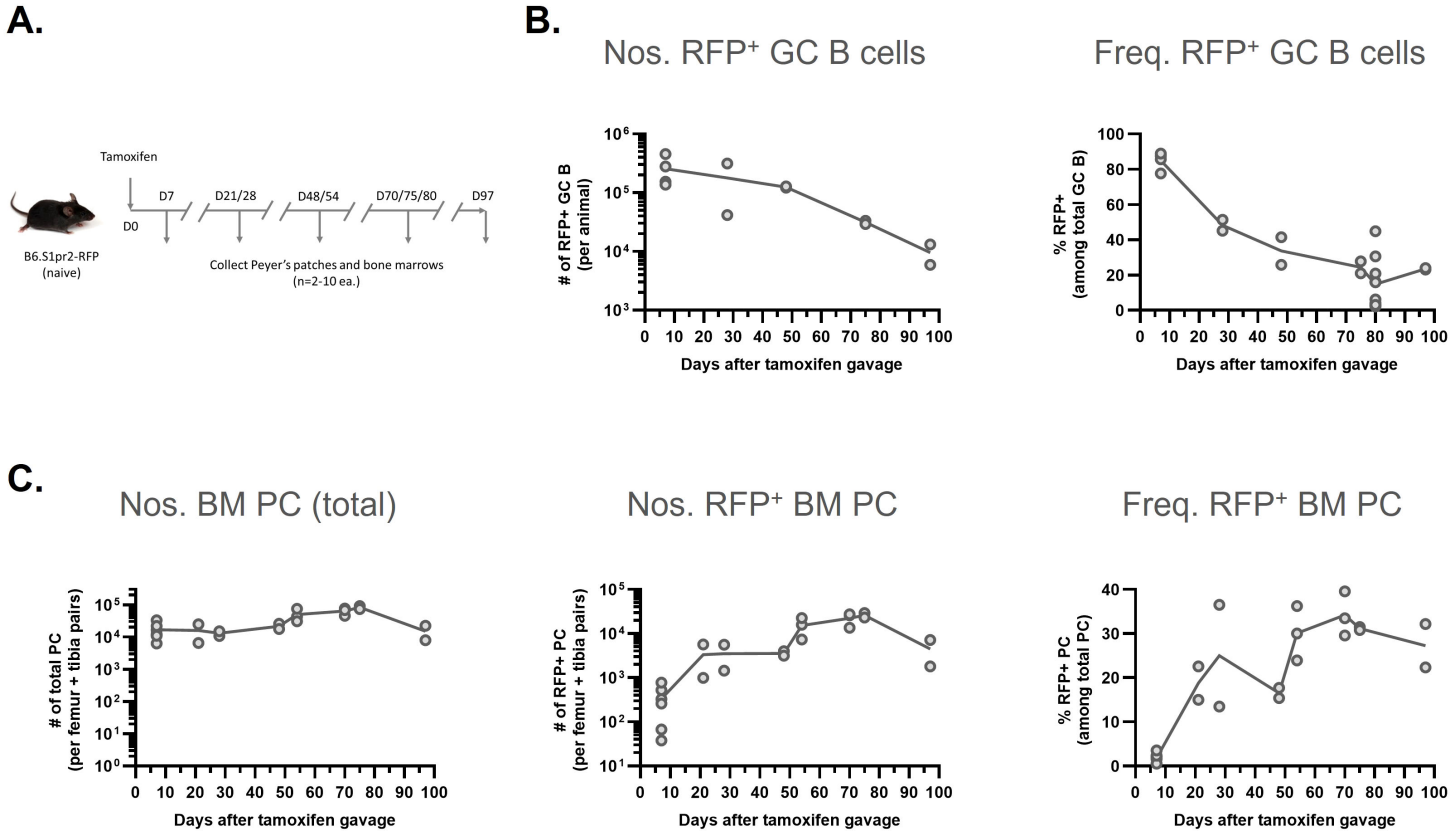
Kinetics of GC B cells in PP and the progenitor PC in BM with RFP labelling. **(A)** Scheme of the experiments is shown. Naïve, 7-12 week old, male and female S1pr2-RFP mice bearing heterozygous of knockin genes (B6 background) were given with tamoxifen orally, and then PP (5-6 nodes per mouse) and BM (femur and tibia pairs from two legs per mouse) were harvested at multiple days (between 7-97, indicated in the figure) after gavage. Each time point has 2-10 replicates of animals tested. Collected samples were prepared to stain surface (both PP and BM) and intracellular (only BM) markers. Enumeration was done by FACSymphony A5. **(B)** Kinetics in number (left) and frequency (right) of RFP^+^ GC B cells in PP are shown. TCRβ^-^ CD138^-^ B220^+^ CD38^lo^ IgD^-^ population was gated as GC B cells. **(C)** Kinetics in number of total (left) or RFP^+^ (center) PC and frequency of RFP^+^ PC (right) in BM are shown. Dump (CD4, CD8a, CD90.2, Gr1, F4/80, Ter119)^-^ CD138^+^ TACI^+^ population was gated as PC. **(B, C)** Each dot represents individual mouse data. The data are pooled from 2-3 independent experiments.

To determine what fraction of BM PC originate from these constitutively active mucosal GCs, we analyzed B6 mice heterozygous for knockin alleles (S1pr2^ERT2Cre^-R26^LSL-dTomato^) that conditionally mark (RFP^+^) GC B cells and their progeny (99). Briefly, naïve 7-12-week-old male and female mice were given Tamoxifen by gavage to pulse label active GC B cells over a period of approximately 60–72 hours (100). Labeled GC B cells and BM PCs were enumerated regularly for over 90 days post-treatment (**Figure 3A**).

Seven days after gavage, some 85% of all Peyer’s patch (5-6/animal) GC B cells expressed the td-Tomato fluorochrome (RFP^+^). With time, the frequencies of RFP^+^ GC B cells fell, to 34% on day 48 and they remained stable from days 75 to 97 at approximately 25%. The loss of RFP^+^ cells represents both migration and differentiation from the GC compartment and the apoptotic losses associated with hypermutation and Tfh selection (27) (**Figure 3B**).

In contrast, BM PC numbers remained generally stable over the sampling period, with a mean ± SEM value of 3.4 (± 0.6) x 10^4^ CD138^+^TACI^+^ cells from femur and tibia pairs from 22 mice (**Figure 3C**). This sample represents approximately 19% of central BM volume in the mouse (95). RFP^+^ cell numbers within the CD138^+^TACI^+^ population rose from a few hundred at 7 days after Tamoxifen gavage to more than 20,000 by day 70. Following this peak, RFP^+^ BM PC numbers fell to approximately 5,000 on day 97. Thus, a single Tamoxifen pulse, which labeled constitutively active GC B cells in mucosal sites, marks PC-fated GC cells in sufficient numbers to account for some 34% of the entire BM PC population in 70–80 days (**Figure 3C**).

**Figure 4:**
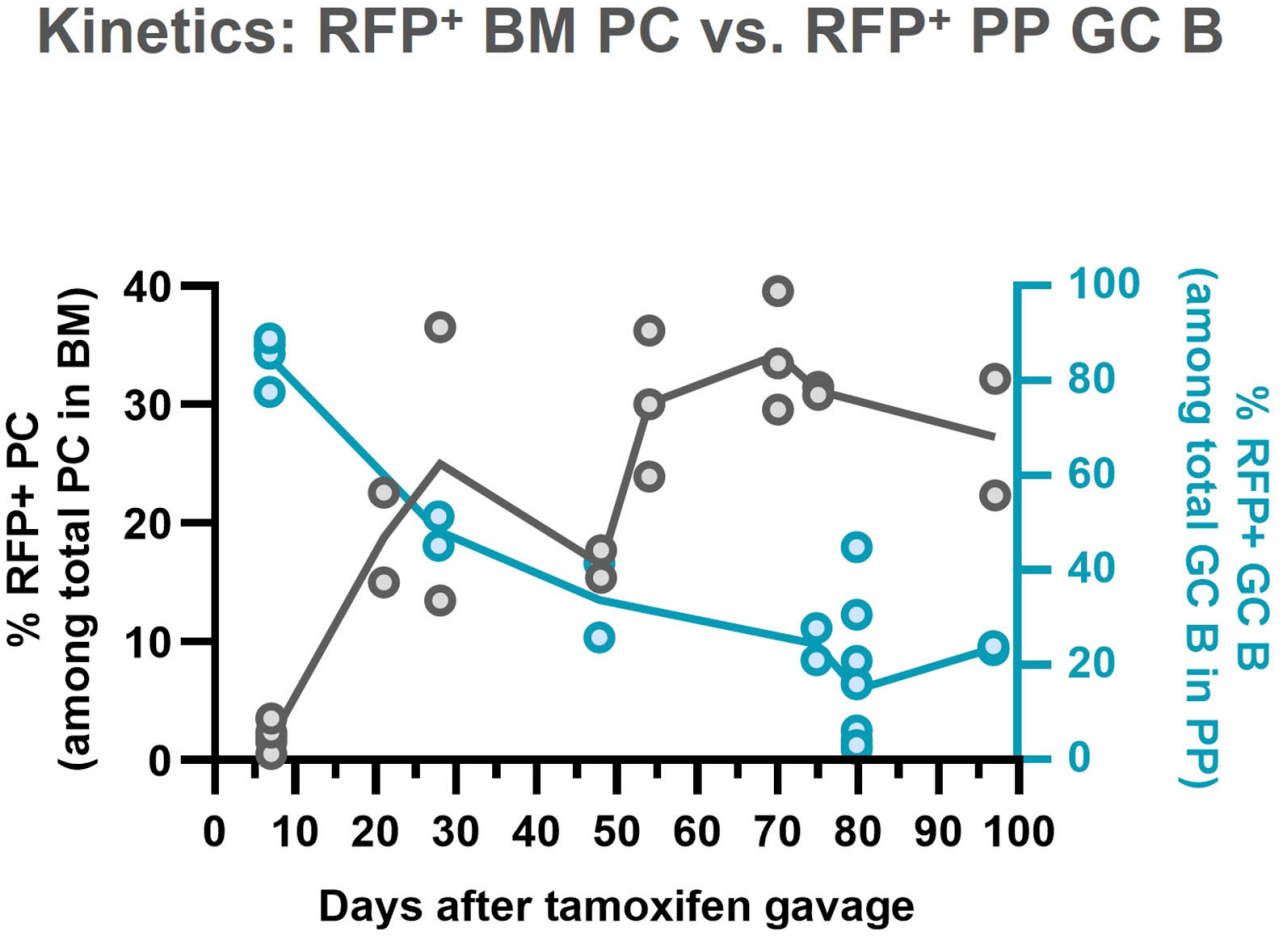
Reciprocal kinetics of RFP^+^-GC B cells in PP and -PC in BM. Figures showing frequencies of RFP^+^ GC B cells in PP and RFP^+^ PC in BM are overlayed. Gray plots and left y-axis correspond to **Figure 1B** (right), and blue plots and right y-axis do **Figure 1C** (right).

The reciprocal behaviors of RFP^+^ GC B cells and BM PC (**Figure 4**) are consistent with the constant BM PC replenishment by constitutively active GC, and with a much-increased survival rate for BM PC versus GC B cells.

To strengthen this correlation, we determined whether the proportions of IgM, IgG, and IgA expression among RFP^+^ and RFP^−^ CD138^+^TACI^+^ BM cells were similar. Similar isotype distributions between labeled and unlabeled BM PC would be consistent with a common origin and that is exactly what we observed (**Table 1**).

**Table 1:** Ig distribution among RFP^+^ and RFP^-^ BM PC.

PC Isotype	Fraction (%) BM CD138^+^TACI^+^ PC			
	Day 7	Day 48	Day 75	Day 97
*RFP^+^ total*	*1 (± 0.1)%*	*17 (± 2)%*	*31 (± 1)%*	*27 (± 7)%*
RFP^+^ IgM	34 (± 13)%	25 (± 13)%	17 (± 12)%	18 (± 8)%
RFP^+^ IgG	8 (± 3)%	12 (± 4)%	3 (± 1)%	8 (± 9)%
RFP^+^ IgA	49 (± 19)%	55 (± 16)%	75 (± 14)%	61 (± 23)%
*RFP* ^−^ *total*	*99 (± 1)%*	*82 (± 2)%*	*67 (± 1)%*	*72 (± 7)%*
RFP^−^ IgM	26 (± 2)%	37 (± 10)%	25 (± 18)%	24 (± 9)%
RFP^−^ IgG	8 (± 2)%	10 (± 1)%	4 (± 2)%	8 (± 8)%
RFP^−^ IgA	52 (± 7)%	44 (± 8)%	63 (± 16)%	58 (± 18)%

On days 7, 48, 75 and 97, BM cells were stained with intracellular Igs (IgA, IgG and IgM) and enumerated by flowcytometry. Dump (CD4, CD8a, CD90.2, Gr1, F4/80, Ter119)^-^ CD138^+^ TACI^+^ population was gated as PC and divided by the RFP expression. Values represent the mean (± SD) frequency of each Ig^+^ cells from two replicated animals at each time point.

We conclude that a great majority of BM PC in naïve mice raised under specific-pathogen free conditions are the products of GC responses to the normal commensal microbial flora. In addition to local and systemic IgA, these responses also generate IgM and IgG BM PC. Consequently, under homeostatic conditions, serum Ab, including serum IgG, is directed toward epitopes present on gut flora, raising the question of whether BM LLPC survival niches are already substantially filled with the progeny of gut-associated GCs. Our fate-mapping experiments provide direct support for this idea by showing that within 70 to 80 days of labeling Peyer’s patch GC B cells, nearly one-third of the BM PC pool can be traced to microbiota-driven GC responses. These results establish that systemic antibody production is strongly shaped by the commensal microbiota. Consistent with this, Liu et al. demonstrated that BM PCs are transcriptionally and functionally heterogeneous, with long-lived subsets arising not only from immunization and autoantigen responses but also in response to the microbiota (43). Importantly, microbiota-derived PCs occupy the same long-lived niches as those induced by vaccination, underscoring that BM survival resources are finite and shared. Together, these findings argue that constitutive GC activity to commensal antigens continuously seeds the BM with long-lived PCs, sustaining steady-state antibody levels while potentially limiting the capacity of vaccine-induced PCs to enter the restricted survival pool. In this regard, it has been shown that HIV envelope (Env)-reactive antibodies in acute HIV infection first derive from microbiota-reactive B cells in the intestine (101) and HIV Env vaccine-induced antibodies are similarly derived from microbiota-reactive B cells (102). It is known that HIV Env B cell responses are quite short-lived (103, 104) implying that pathogen-targeted PC responses may be limited in their ability to compete with microbiota-targeted precursors for BM niches.

### Conclusion and future perspectives

The significance of gut microbiota on both the intestinal and systemic immune systems, is now widely appreciated. This influence is mediated through several mechanisms, including translocation of microbes and microbial products leading to distal hematopoietic effects and changed immunological “tone” (105). Humoral immunity is impacted by gut microbiota through modulating local B-lymphopoiesis (106) and by influence on the primary Ig/BCR repertoire (107). We now propose that these microbiota effects extend to the BM PC populations typically responsible for serum Ab durability and, potentially, to competition for LLPC survival niches.

LLPCs form the cellular basis of durable humoral immunity, yet the mechanisms that govern their induction, maintenance, and systemic integration remain incompletely defined. While both intrinsic differentiation programs and extrinsic survival niches clearly contribute, the field still lacks a coherent framework for how SLPCs acquire longevity, why some antigens elicit LLPCs while others do not, and how survival niches are organized, remodeled, and shared between microbiota-driven and vaccine-induced responses.

In homeostasis, we calculate that 2.0 – 3.7x10^6^ PC are sufficient to maintain serum IgG levels, PC numbers significantly greater than can be recovered from BM. LLPC populations can also be recovered from gut and intestine of animals and humans (4, 8, 96) and it is technically straightforward to enumerate PC numbers in diverse tissues to estimate better the niche capacity for IgG PC. This accounting exercise will be important in determining the roles for these tissues in systemic humoral protection. It will also be critical to determine the capacity of this survival niche through the lifetime of the animal and how it may respond to disease. We note that even modest levels of inflammatory stimuli can have profound effects on BM cell populations (108–110).

Importantly, most mechanistic insights derive from murine systems. Recent high-dimensional profiling of human BM PCs has provided a framework to distinguish bona fide LLPCs from shorter-lived subsets. In a seminal study, Nguyen et al. used flow cytometry and single-cell transcriptomics to define multiple BM PC populations (Population A to D). Population D (PopD) cells represented canonical LLPCs, characterized by loss of CD19, low CD45 expression, a distinct metabolic and pro-survival transcriptional program, and long-term persistence (94, 111). By contrast, PopA to C displayed phenotypic features of more recently generated or transitional PCs with less pronounced survival signatures (94). This framework establishes a human reference point for LLPC versus non-LLPC phenotypes, providing an essential benchmark to interpret murine models and guiding efforts to determine how BM niches selectively maintain PopD cells.

Outstanding questions include whether GC- and extrafollicular pathways yield functionally distinct LLPCs, whether continued Tfh input is required for their maturation, how PC pools are replenished after depletion, what role an underexplored “homeostatic pool” of memory B cells plays, how faithfully murine models capture LLPC biology in humans and nonhuman primates, and what numbers and qualities of LLPCs across tissues are needed to sustain lifelong antibody homeostasis. Closing these gaps will require integration of single-cell multi-omics, advanced *in vivo* imaging, and functional perturbation of niche components across species. Such approaches can clarify how LLPC fate is specified, how BM and mucosal niches adapt over time, and how PC pools are replenished and maintained.

Beyond their basic immunological importance, these insights carry direct translational weight. By aligning murine mechanistic models with the human PopD framework, the field can better define the principles of LLPC maturation and persistence, thereby guiding the rational design of vaccines that promote decades-long protection, informing strategies to sustain protective antibody levels in immunocompromised individuals, and suggesting therapeutic approaches to selectively eliminate autoreactive LLPCs in autoimmunity. A deeper mechanistic understanding of LLPC biology will not only close longstanding conceptual gaps but also open new opportunities to engineer humoral immunity that is both potent and enduring.

## Materials and methods

### Mice and Tamoxifen gavage

S1pr2-ERT2^cre^-tdTomato mice provided by T. Kurosaki, were maintained under specific pathogen–free conditions at the Duke University Animal Care Facility. Male and female (7–12 weeks old) mice were given 12.5 mg of Tamoxifen (Sigma-Aldrich) in 250 μl of corn oil by gavage. BM and/or PP tissue was collected from treated mice 7–97 days after Tamoxifen administration for enumeration and characterization PCs/ASCs and GC B cells, respectively. These experiments were approved by the Duke University Institutional Animal Care and Use Committee.

### ELISpot assay

ELISpot plates (Millipore) were coated with goat anti-mouse Igκ + Igλ antibodies (2 μg/ml each; Southern Biotech) in carbonate buffer (pH 9.0), blocked with PBS containing 0.5% BSA, and then applied with serially-diluted BM cells (5.0x10^4^ – 3.1x10^3^ cells/well). After one hour incubation in a CO_2_ incubator at 37 °C, plates were blocked again, and AP-conjugated goat anti-mouse Ig secondary antibody (anti-IgM, -IgG or -IgA; 1:10,000; Southern Biotech) was added for another 1 hour. Spots were visualized by BCIP-NBT (Sigma) and counted by a CTL ImmunoSpot S6 instrument.

### Flow cytometry

BM was obtained by flushing tibia and femur pairs of both legs and lysed with ACK. PPs were collected from small intestine (5–6 nodes/animal) and single-cell suspensions were made by gentle disruption between glass slides. All of the cells were suspended in media and pre-treated with the mixture of anti-CD16/CD32 Ab (2.4G2) and rat IgG (Invitrogen). BM cells were then incubated with biotin-conjugated antibodies: anti-CD4 (GK1.5), -CD8α (53-6.7), -CD90.2 (30-H12), -Gr1 (RB6-8C5), -Ter119 (TER-119) and -F4/80 (BM8); BioLegend), followed by the incubation with streptavidin microbeads (Miltenyi) for MACS depletion of non-B cell lineages. Surface staining was by fluorophore-conjugated antibodies to: TCR β-chain (H57-597), B220 (RA3-6B2), CD38 (90), CD138 (281-2), IgD (11-26c.2a), and TACI (8F10). For intracellular staining, cells were fixed and permeabilized by BD Pharmingen™ Transcription Factor Buffer Set (BD) and labeled with fluorophore-conjugated antibodies to: IgM (II/41), IgG1 (A85-1), IgG2a/b (R2-40), IgG3 (R40-82), and IgA (C10-3). GC B cells were identified as the TCRβ^-^ CD138^-^ B220^+^ CD38^lo^ IgD^-^ population. PCs were identified as the Dump (CD4, CD8a, CD90.2, Gr1, F4/80, Ter119)^-^ CD138^+^ TACI^+^ population. Cell doublets were excluded by FSC-A/FSC-H gating and dead cells by signal. Cell populations characterized in a BD FACSymphony A5 instrument and analyzed by FlowJo software (Treestar Inc.).

## Data Availability

The raw data supporting the conclusions of this article will be made available by the authors, without undue reservation.
